# Thyroid Status During Pregnancy in Women With Polycystic Ovary Syndrome and the Effect of Metformin

**DOI:** 10.3389/fendo.2022.772801

**Published:** 2022-02-21

**Authors:** Anastasia Trouva, Michael Alvarsson, Jan Calissendorff, Bjørn Olav Åsvold, Eszter Vanky, Angelica Lindén Hirschberg

**Affiliations:** ^1^ Department of Molecular Medicine and Surgery, Karolinska Institutet, Stockholm, Sweden; ^2^ Department of Internal Medicine, Section of Diabetes and Endocrinology, Södersjukhuset, Stockholm, Sweden; ^3^ Kristian Gerhard (K.G.) Jebsen Center for Genetic Epidemiology, Department of Public Health and Nursing, Norges Teknisk-Naturvitenskapelige Universitet (NTNU), Norwegian University of Science and Technology, Trondheim, Norway; ^4^ Helseundersøkelsen i Nord-Trøndelag (HUNT) Research Center, Department of Public Health and Nursing, Norges Teknisk-Naturvitenskapelige Universitet (NTNU), Norwegian University of Science and Technology, Levanger, Norway; ^5^ Department of Endocrinology, Clinic of Medicine, St. Olavs Hospital, Trondheim University Hospital, Trondheim, Norway; ^6^ Department of Clinical and Molecular Medicine, Faculty of Medicine and Health Sciences, Norwegian University of Science and Technology, Trondheim, Norway; ^7^ Department of Obstetrics and Gynaecology, St. Olavs Hospital, Trondheim University Hospital, Trondheim, Norway; ^8^ Department of Women’s and Children’s Health, Karolinska Institutet, Stockholm, Sweden; ^9^ Department of Gynecology and Reproductive Medicine, Karolinska University Hospital, Stockholm, Sweden

**Keywords:** hypothyroidism, PCOS (polycystic ovarian syndrome), pregnancy, pregnancy outcome, metformin

## Abstract

**Objective:**

Polycystic ovary syndrome (PCOS) and hypothyroidism are related conditions, and both are associated with adverse pregnancy outcomes. Knowledge is lacking about the complex interaction between thyroid status and PCOS during pregnancy. We investigated the thyroid status and its association with pregnancy complications in PCOS, and in relation to metformin treatment.

**Design:**

*Post-hoc* analyses of two randomized, double-blind, placebo-controlled trials.

**Methods:**

288 pregnant women with PCOS were randomized to treatment with metformin or placebo from first trimester to delivery. We measured serum levels of thyroid stimulating hormone (TSH) and free thyroxine (fT4) at gestational week (gw) 5-12, 19, 32 and 36 and related to metformin treatment and pregnancy complications. Thyroid peroxidase antibodies (TPO-ab) were analyzed at inclusion and at gw 36.

**Results:**

The overall prevalence of subclinical and overt hypothyroidism was 1.5% and 0%, respectively. The TSH level was not affected by metformin, whereas fT4 was significantly higher in the metformin group with less decrease throughout pregnancy compared to placebo, p<0.001. A lower decrease in fT4 during pregnancy correlated to less weight gain (r= -0.17, p=0.020) and tended to be associated with reduced odds ratio for gestational diabetes (OR 0.85 per 1 pmol/L, 95% CI 0.71;1.02).

**Conclusions:**

In women with PCOS, metformin treatment during pregnancy was associated with less decrease in fT4 compared to placebo, while it did not affect TSH. A smaller decrease in fT4 correlated to less weight gain and tended to be associated with a lower risk of gestational diabetes.

**Clinical Trial Registration:**

ClinicalTrials.gov, identifier NCT00159536 (The PregMet study); identifier NCT03259919 (The pilot study).

## Introduction

Polycystic ovary syndrome (PCOS) affects up to 13% of women in reproductive age (1). Insulin resistance plays a pivotal role in the etiology of the syndrome which is closely linked to both metabolic and reproductive abnormalities (2). PCOS is also related to thyroid dysfunction. In a cross-sectional study by Novais et al. the prevalence of subclinical hypothyroidism (SCH) in non-pregnant women with PCOS was 16.9% compared to 6.2% in the non-PCOS group (3). Autoimmune thyroiditis is also reported to be more prevalent in non-pregnant PCOS women compared to controls (4, 5).

Pregnant women with PCOS are at an increased risk for complications such as gestational diabetes (GDM), preterm birth, delivery by caesarian section, low birth weight and admission of the neonate to the intensive care unit (6). Likewise, overt hypothyroidism (OH) as well as borderline thyroid dysfunction during pregnancy have been associated with miscarriage, preterm delivery, low birthweight, preeclampsia (PE) and GDM (7), as well as adverse neonatal outcome (8, 9). Furthermore, the occurrence of thyroid peroxidase antibodies (TPO-ab) as such has been linked to miscarriage and preterm delivery (10, 11). According to a recent register-based study from Denmark, the OR for thyroid disease (based on diagnosis coding) in women with PCOS during pregnancy and one year post-partum was 2.3 *vs* controls (12). However, the prevalence of thyroid dysfunction in pregnant women with PCOS is not known, and neither is the impact of thyroid function on pregnancy complications in these women.

Metformin, a commonly used insulin-sensitizing drug, has been used in several studies to investigate pregnancy complications in women with PCOS (13–15). A recent pooled analysis of individual participant data from three randomized controlled trials (RCTs) showed a significant reduction of late miscarriages and preterm births in metformin treated women with PCOS compared to placebo, but no effect of metformin was observed on PE and GDM (16). The effect of metformin on thyroid function has been the subject of interest in several studies. Investigations in non-pregnant women with PCOS and hypothyroidism, point towards a thyroid stimulating hormone (TSH)-lowering effect of metformin (17, 18). It is unclear how metformin may affect the thyroid hormone status of women with PCOS during pregnancy.

The present study is a *post-hoc* analysis of two RCTs on the effect of metformin on pregnancy complications in women with PCOS. Our aim was to investigate thyroid hormone status in relation to metformin treatment and its association with pregnancy complications in women with PCOS.

## Materials and Methods

### Study Design

**Figure 1:**
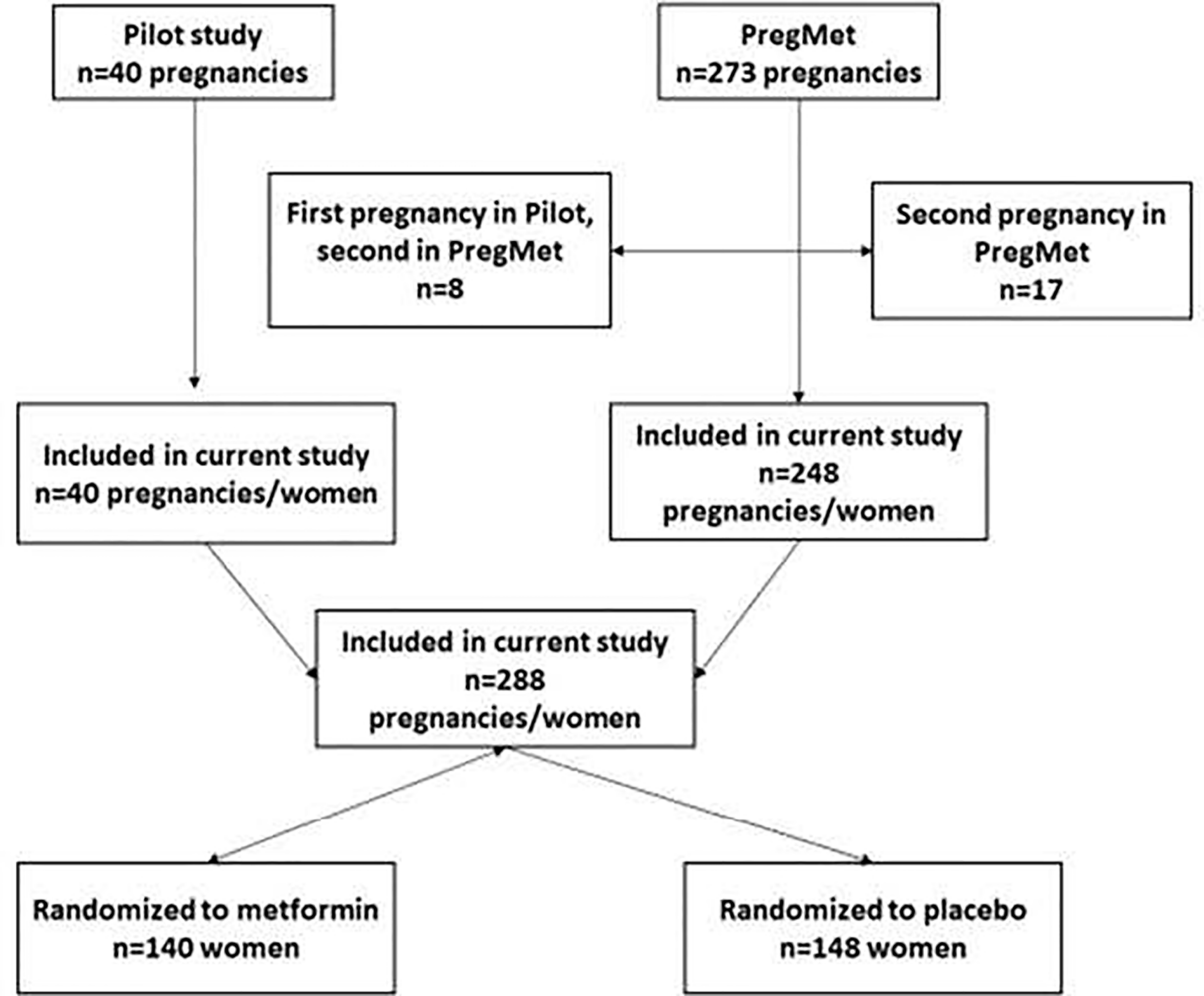
Flowchart of the study design.

The current study is a *post-hoc* analysis of two randomized, controlled, double-blinded studies; the pilot and the PregMet study (**Figure 1**) (19, 20). In both studies, pregnant women with PCOS were randomized to either metformin or placebo to assess the potential of metformin to prevent pregnancy complications.

The Committee for Medical Research Ethics of Health Region IV, Norway, approved the pilot study (project number 51-2000), the PregMet study (project number 145.04) and the supplement application for the current study (reference number 2010/3115 145-04).

### The Pilot Study

40 women with PCOS were included at St Olav’s Hospital in Trondheim, Norway from 2000-2003. All women met the Rotterdam 2003 criteria for PCOS in a retrospect analysis (21). Inclusion criteria for the pilot study were: (1) PCOS diagnosed prior to the index pregnancy; (2) age 18 to 40 years; (3) gestational age between 5 and 12 weeks; and (4) a singleton viable fetus. The participants were randomized to metformin 1700 mg daily or placebo (19).

### The PregMet Study

In total, 257 women with PCOS and 273 pregnancies were included from 11 study centers in Norway. Inclusion criteria for the PregMet study were: (1) PCOS diagnosed according to the Rotterdam criteria (21); (2) age 18 to 45 years; (3) gestational age between 5 and 12 weeks; and (4) a singleton viable fetus. The participants were randomized to either 2000 mg metformin daily or placebo (20).

In both the pilot and the PregMet studies, an intake of more than 85% of the prescribed tablets was self-reported by 80% of the participants, which were thus considered to have good/acceptable compliance. Fasting blood samples were drawn at inclusion (gestational week (gw) 5-12), gw 19 ± 1, gw 32 ± 1, and at gw 36 ± 1. Randomization, blinding, participant flow, and measurements performed in the pilot and the PregMet studies are described in detail elsewhere (19, 20).

### The Present Study

The flowchart of randomization, dropouts and exclusions are shown in **Figure 1**. Among the participants in the pilot and the PregMet study there were 8 and 17 respectively who participated with two pregnancies, i. e. first in the pilot and then in the PregMet study or twice in the PregMet study. For the current study, we decided to include only one pregnancy from each woman, in this case only the first one. In total, 288 women with one pregnancy were included in our analyses.

We measured TSH and free thyroxine (fT4) from blood samples at inclusion, gw 19, 32 and 36. For the analysis of TPO-ab, we used blood samples collected at inclusion and gw 36. All blood samples were stored at -70-80°C. In order to calculate the prevalence of hypothyroidism in the beginning of the pregnancy, we analyzed the thyroid status of the participants with no former history of thyroid disorders, as there was a group of women who had already been diagnosed with hypothyroidism prior to inclusion and were being treated with levothyroxine (n=13). We calculated the total prevalence of “any kind of hypothyroidism” using three groups: the group of patients with a diagnosis of hypothyroidism prior to inclusion, the “SCH group” and the “OH group”. All participants were included in the mixed model analysis regarding the effect of metformin treatment.

We furthermore investigated if changes in TSH and fT4 could predict pregnancy outcomes including preterm delivery (spontaneous or operative birth occurring before gestational week 37 + 0), preeclampsia [blood pressure measured after at least 10 minutes of comfortable chair rest ≥140/90 mm Hg at two different occasions and proteinuria (1+ protein in two different occasions or 2+ protein in one occasion on a urinary dip stick)] and GDM [fasting plasma glucose ≥7.0 mmol/L or a 2 hour plasma glucose ≥ 7.8 mmol/L during a standard 75 g per oral glucose tolerance test (OGTT) performed at inclusion, gw 19 and 32 according to the WHO 1999 standard].

### Laboratory Analysis

Serum levels of TSH, fT4 and TPO-ab were determined at the Research Laboratory of Women’s and Children’s Health Unit, Karolinska University Hospital, Stockholm, Sweden by chemiluminescent enzyme immunometric assays using commercial kits procured from Siemens Medical Solutions Diagnostics (fT4 and TPO-ab) and Diagnostic Products Corporation (TSH) (Immulite^®^, Los Angeles, CA). TPO-ab was considered positive if the value was ≥ 35 IU/mL. Respective detection limits (and intra-assay and inter-assay coefficients of variation) were as follows: for TSH 0.004 mIU/L (5.0% and 9.9%), for fT4 3.6 pmol/L (4.5% and 5.5%) and for TPO-ab 7 IU/mL (4.3% and 10.5%). The method-specific reference range provided by the manufacturer was 0.4 – 4 mIU/L for TSH and 11.5 – 22.7 pmol/L for fT4.

### Definitions of Thyroid Hormone Status

In order to calculate the prevalence of hypothyroidism in the first trimester of pregnancy we applied the trimester-specific reference range for TSH recommended by ATA 2017 (22), which is 0.1-4 mIU/L. For fT4 we used the method-specific reference range provided by the manufacturer, which is used clinically for non-pregnant subjects, due to lack of trimester-specific ranges.

Euthyroidism: Levels of TSH and fT4 within the reference range as above.

SCH: TSH of 4.1-9.9 mIIU/L in combination with fT4 within the reference range.

OH: TSH above the reference range in combination with fT4 below the reference range as well as TSH levels of 10.0 mIU/L or above, irrespective of fT4 levels.

Isolated hypothyroxinemia: TSH within the reference range and fT4 below the reference range.

Subclinical hyperthyroidism: TSH below the reference range in combination with fT4 within the reference range.

Overt hyperthyroidism: TSH below the reference range in combination with fT4 above the reference range.

### Statistical Analysis

Continuous data are presented as mean and standard deviation (SD), and categorical data are summarized using counts and percentages. The prevalence of hypothyroidism at baseline is presented as percentage with a 95% confidence interval (CI). Repeated measurements of thyroid status (TSH, fT4) during pregnancy were analyzed using a mixed model of the following for: Y_ijk_ = intercept + Ti + W_j(i)_ + GW_k_ + T*GW_ik_ + ε

Y_ijk_ = the observed continuous variable (fT4 and TSH)

T_i_ = treatment i (placebo and metformin), fixed factor.

W_j(i)_ = women j within treatment i.

GW_k_ = gestational week k (5-12, 19, 32 and 36), fixed factor. The covariance structure for the four repeated measurements is unstructured (UN). In the unstructured covariance pattern, the variances of responses differ for each time period, and the covariances differ between each pair of time periods.

T*GW_ik_ = interaction between treatment and gestational week, fixed factor.

ε = residual

In case of significant interaction, the effect of one factor was tested keeping the other factor fixed. Bonferroni corrections of the p-values were then performed when appropriate. Prior to these analyses, fT4 was log-transformed and TSH was squared root transformed due to positive skewness (23). Graphically, antilog values of fT4 are presented with geometric mean and 95% confidence interval and TSH with median and quartile range as back-transformation for square root transformation cannot provide meaningful confidence intervals. Repeated measurements of TPO-ab, divided in two classes (<35, ≥35) during pregnancy from inclusion (gw 5-12) to gw 36, were analyzed using generalized estimating equation (GEE, GENLIN in SPSS), with the same factors as in the mixed model. In this case, the distribution of the outcome variable is binomial with a logit link function. The covariance structure for the two repeated measurements is exchangeable (compound symmetry). The Mixed Model and the GEE procedures in SPSS handle missing data. The effect of the treatments and the change in fT4 and TSH from baseline to gw 19 were investigated as possible predictors of the pregnancy outcomes preeclampsia, premature delivery and GDM, using multiple logistic regressions, one for each outcome. The interaction between the treatments and the change in fT4 and TSH was included in the logistic regression model according to the following;


$$\lambda =\text{intercept}+\beta_{1}*\text{T}+\beta_{2}*\text{X}+\beta_{3}*\text{T}*\text{X}.$$

λ=^e^log(probability of one pregnancy outcome/1- probability of one pregnancy outcome).

T = the treatment placebo (0) and metformin (1).

X= a continuous variable (change in fT4 and change in TSH).

T*X = the interaction between treatment and the continuous variable.

However, no interaction effects could be demonstrated for any of the outcome variables and therefore the main factors only were kept in the final models. These analyses were also performed with adjustment for age and BMI. The Spearman Rank correlation was calculated to measure the correlation between the weight gain and the change in fT4 and TSH from baseline to gw 36. Analyses of covariance, ANCOVA, was used to test whether the weight gain during pregnancy could explain the relationship between the treatment groups and the change in fT4 from baseline to gw 36 according to the following:


$${\text{Y}}_{\text{ij}}=\text{intercept}+\text{Ti}+\beta *{\text{X}}_{\text{j}}+\varepsilon$$

Y_ij_ = the observed change in fT4.

T_i_ = treatment i (placebo and metformin).

X_j_ = weight gain for patient j within treatment

ε = residual.

In all analyses a p-value of less than 0.05 was considered statistically significant.

Statistical calculations were done with the statistical program IBM Corp. Released 2019. IBM SPSS Statistics for Windows, Version 26.0. Armonk, NY: IBM Corp and Statistica for Windows 13.5 (TIBCO Software Inc. USA.

## Results

Baseline demographic characteristics and clinical parameters were comparable between the metformin and the placebo groups (**Table 1**).

**Table 1:** Baseline characteristics of participants.

	Total n = 288	Metformin n = 140	Placebo n = 148
*Demographics*			
Age (years)	29.1 ± 4.4	29.4 ± 4.5	28.9 ± 4.3
Weight (kg)	80.8 ± 18.8	82.2 ± 19.1	79.5 ± 18.5
BMI (kg/m^2^)	28.8 ± 6.6	29.4 ± 6.6	28.3 ± 6.6
Parity (%)			
0	61.5	63.6	59.5
1	30.6	30.7	30.4
2	6.6	4.3	8.8
3	1.4	1.4	1.4
Smoking (%)	8.1	10.7	5.6
Caucasian ethnicity (%)	97.6	96.4	98.6
Hypothyroidism prior to inclusion (%)	4.5	4.3	4.7
Metformin treatment at conception (%)	30.6	31.4	29.7
Gestational age at inclusion (days)	73 ± 14	73 ± 14	72 ± 14
Positive TPO-ab (%)	7.1	7.4	6.9
			
*Clinical characteristics*			
Systolic blood pressure (mm Hg)	118 ± 12	119 ± 13	117 ± 12
Diastolic blood pressure (mm Hg)	73 ± 11	74 ± 12	72 ± 10
Glucose fasting (mmol/L)	4.6 ± 0.5	4.6 ± 0.5	4.7 ± 0.6
Glucose 2h (mmol/L)	5.7 ± 1.6	5.6 ± 1.5	5.8 ± 1.7

Data presented as mean ± SD or n (%) as appropriate.

None of the comparisons between the groups showed statistically significant difference (p-value < 0.05).

**Figure 2:**
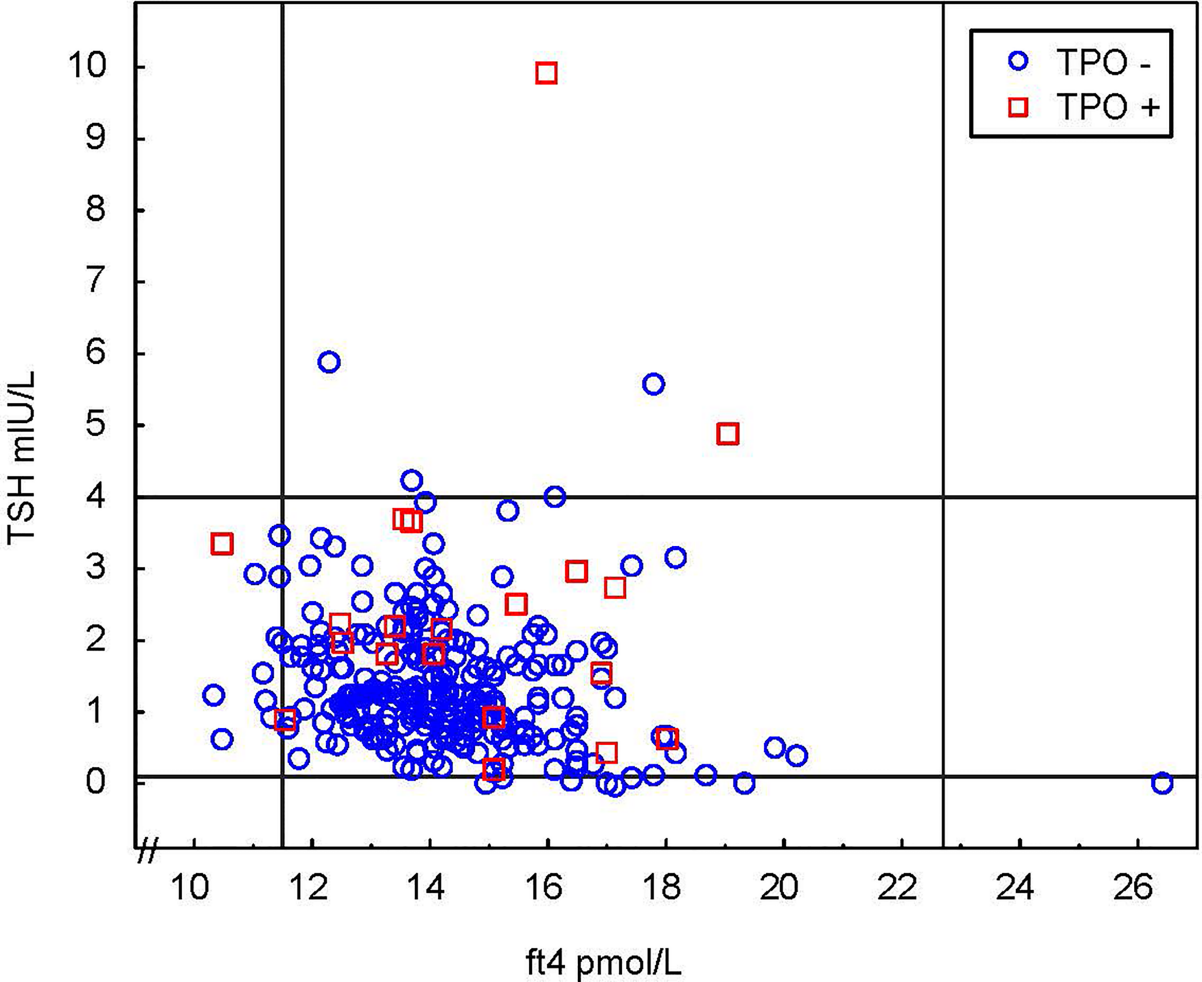
Thyroid status of all participants at inclusion in 1st trimester. Continuous lines represent the first trimester reference range recommended by ATA 2017 for TSH and reference limits for non-pregnant individuals for fT4.

Prior to inclusion, 13 women (4.5%) had a diagnosed and treated hypothyroidism and no one had hyperthyroidism. The thyroid hormone status (TSH, fT4 and TPO-ab) at inclusion in women with no previously known hypothyroidism is shown in **Figure 2**. Using a TSH cut-off of 4 mIU/L, the prevalence at inclusion of undiagnosed SCH and OH was 1.5% (95% CI: 0.58;3.77) and 0% (95% CI: 0.00; 1.41) respectively (**Table 2**). The prevalence of known and undiagnosed hypothyroidism combined was (4 SCH) + (0 OH) + (13 known) = 17/281 = 6%. There are missing data on seven subjects. **Table 2** also presents data on isolated hypothyroxinemia and hyperthyroidism.

**Table 2:** Prevalence of euthyroidism, hypo- and hyperthyroidism at first trimester.

	N	% (95% CI)	n, TPO-ab (–)	n, TPO-ab (+)
**Euthyroidism**	246	91.8 (87.9; 94.5)	233	13
**Hypothyroidism**				
Subclinical hypothyroidism	4	1.5 (0.6; 3.8)	4	0
Overt hypothyroidism	0	0 (0.0; 1.4)		
Isolated hypothyroxinemia	11	4.1 (2.3; 7.2)	10	1
**Hyperthyroidism**				
Subclinical hyperthyroidism	1	0.4 (0.1; 2.1)	1	0
Overt hyperthyroidism	6	2.2 (1.0; 4.8)	6	0

Out of 288 women, 13 were diagnosed and treated for hypothyroidism prior to inclusion and 7 had missing data, resulting in 268 women included in this analysis.

**Figure 3:**
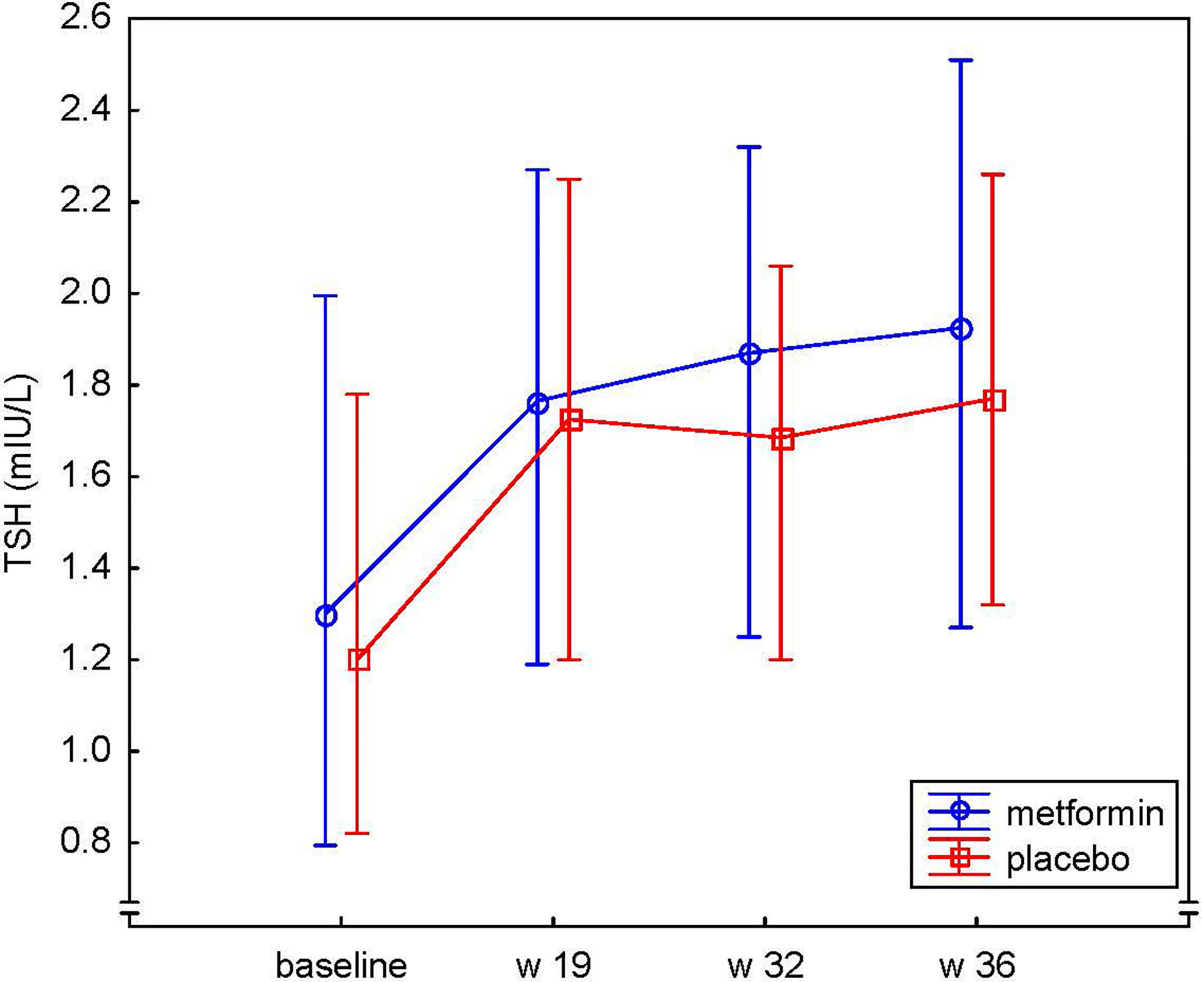
Serum levels of TSH (mIU/L) at baseline, gestational week 19, 32 and 36 in the metformin and placebo groups presented as medians and 25^th^-75^th^ percentiles.

**Figure 4:**
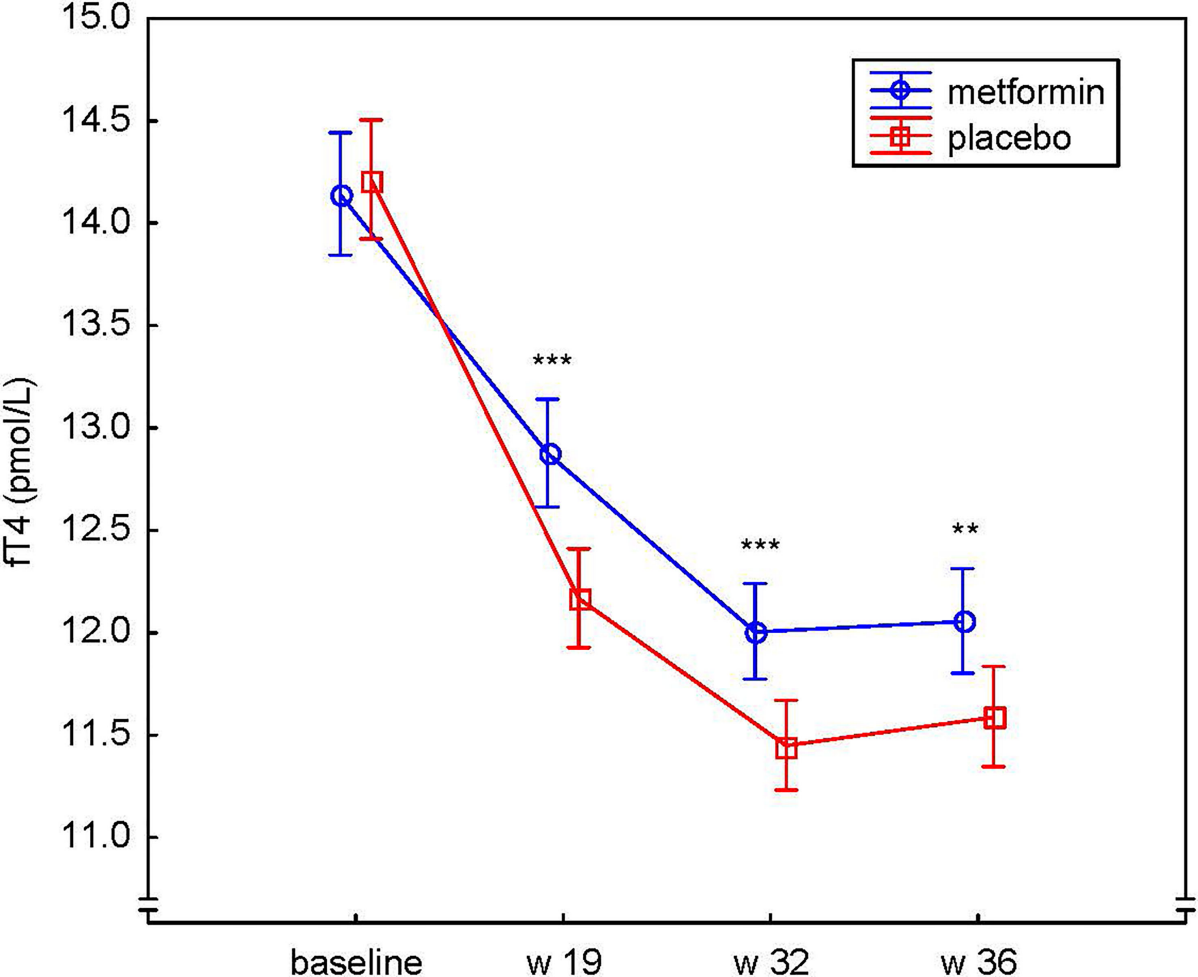
Serum levels of fT4 (pmol/L) at baseline, gestational week 19, 32 and 36 in the metformin and placebo groups presented as geometric means and their 95% confidence intervals. **p < 0.01, ***p < 0.001.

There was no evidence of difference in the mean level of TSH between the metformin and placebo groups at any time point during pregnancy (**Figure 3**). However, a significant difference was observed between the groups regarding the change of fT4 over time, (the treatment * week interaction), p<0.001 (**Figure 4**). The mean level of fT4 was significantly higher (although within the reference range) in the metformin group compared to the placebo group (overall p<0.001) and specifically at gw 19 (p<0.001), gw 32 (p=0.001) and gw 36 (p=0.01). Furthermore, we could demonstrate a difference between the groups regarding the change in fT4 from baseline to different time points throughout pregnancy, i.e. fT4 decreased less in the metformin group (**Figure 4**). The mean differences, in the log scale log(pmol/L), between the groups (metformin – placebo) regarding the change from baseline were: to gw 19 -0.06, 95% CI (-0.09;-0.04), p<0.001, to gw 32 -0.05, 95% CI (-0.08;-0.02), p<0.001, to gw 36 -0.04, 95% CI (-0.07;-0.01), p=0.004.

Gestational weight gain was significantly lower in the metformin group compared to placebo (9.2 ± 5.2 *vs* 11.6 ± 11.1 kg, p=0.02), as previously shown (19, 20). There was a weak negative correlation between gestational weight gain and the decrease of fT4 from baseline to gw 36 in the two groups combined, i.e. the less weight gain, the smaller the decrease in fT4 (r = -0.17, p=0.020). However, the difference between the metformin and placebo groups regarding the change in fT4 between inclusion and gw 36 was independent of the change in weight during the same period [unadjusted 0.49 pmol/L 95% CI (0.07;0.91) and adjusted for weight change 0.46 pmol/L 95% CI (0.03;0.88)], i. e, the weight change during pregnancy could not explain the difference in fT4 between the metformin and placebo groups.

The prevalence of TPO-ab positivity was similar between the groups at inclusion and at the end of pregnancy (7.4% and 4.9% in the metformin group *vs* 6.9% and 6.0% in the placebo group, overall p=0.66). Furthermore, the change in TPO-ab (decrease, no change or increase) during pregnancy was similar between the groups (p=0.20).

**Table 3** shows pregnancy outcomes in the metformin and placebo groups, respectively. As previously reported (24), we found a significantly lower risk of preterm delivery in the metformin group compared to placebo (4% and 11% respectively, p=0.02) whereas there was no difference for gestational diabetes and preeclampsia. Logistic regression showed a trend of an association between the change in fT4 from inclusion to gw 19 and the outcome GDM (OR 0.85 per 1 pmol/L, 95% CI (0.71;1.02), p= 0.07), i.e. the odds for GDM tended to be lower if the decrease in fT4 from the first to the second trimester was less profound. Adjustment for age and BMI did not change this association (**Table 4**). There was no statistically significant association between the change in fT4 and the other outcomes, i.e. preterm delivery and preeclampsia. Furthermore, the change in TSH between inclusion and gw 19 was not associated with these pregnancy complications.

**Table 3:** Pregnancy outcome.

	Metformin	Placebo	p value
	[n (%)]	[n (%)]	
**Preterm Delivery**	5/140 (4)	16/147 (11)	0.02
**Preeclampsia**	12/140 (9)	7/148 (5)	0.19
**Gestational Diabetes Mellitus**	24/140 (26)	23/147 (28)	0.73

**Table 4:** Odds Ratios and 95% CI for pregnancy complications per 1 mIU/L increase in TSH and per 1 pmol/L decrease in fT4 between inclusion and gw 19, estimated from logistic regression models with and without adjustment for age and BMI.

	Treatment	Treatment + Age	Treatment + BMI	Treatment	Treatment + Age	Treatment + BMI
	Change in TSH			Change in fT4		
**Preterm Delivery**	1.28 (0.75; 2.18)	1.34 (0.77; 2.33)	1.30 (0.76; 2.23)	0.98 (0.76; 1.26)	0.96 (0.75; 1.24)	0.97 (0.75; 1.25)
**Preeclampsia**	0.82 (0.58; 1.17)	0.83 (0.58; 1.19)	0.83 (0.57; 1.19)	1.07 (0.77; 1.49)	1.07 (0.76; 1.50)	1.02 (0.74; 1.42)
**Gestational Diabetes Mellitus**	0.92 (0.69; 1.24)	0.92 (0.69; 1.24)	0.92 (0.69; 1.24)	0.85 (0.71; 1.02)	0.85 (0.71; 1.02)	0.84 (0.70; 1.01)

## Discussion

According to our best knowledge, this is the first study on thyroid hormone status throughout pregnancy in women with PCOS treated with metformin or placebo. Metformin did not affect the level of TSH during pregnancy compared to placebo. However, the decrease in fT4 was smaller in the metformin group resulting in higher normal levels of fT4 throughout the course of pregnancy. Furthermore, a smaller decrease in fT4 tended to be associated with a lower risk of GDM.

Using a TSH cut-off of 4 mIU/L according to ATA 2017 recommendations (22), the prevalence of SCH and OH in early pregnancy in the women with PCOS was 1.5% and 0% respectively. Our results are in agreement with the prevalence in 1416 non-PCOS women in early pregnancy reported by Karcaaltincaba et al. (2020), who estimated the prevalence of SCH and OH to be 2.3% and 0.6% respectively, using the same TSH cut-off level (25). However, the prevalence of SCH in the present study is considerably lower when compared to studies in non-pregnant women with PCOS. Thus, in the meta-analysis of Ding et al. (2018) the prevalence of SCH (using a TSH cut-off of 2.5-5 mIU/L) varied between 11.3% and 36.6% with an increased OR of 2.87 compared to controls (4). A potential explanation to the discrepant results could be differences in age and BMI of the various populations studied, besides different cut-off levels of TSH. Our study population consisted of relatively young women with average BMI of 29 and their pregnant status could be considered as a sign of good reproductive and metabolic health.

In the present study, the TSH level during pregnancy was comparable between the metformin and the placebo group. In a meta-analysis of Lupoli et al. (2014), it was demonstrated that metformin induced a reduction in TSH levels in patients with diabetes/insulin resistance with OH and SCH but not in euthyroid subjects (26). Furthermore, Rotondi et al. (2011) found a TSH-lowering effect of metformin in non-pregnant women with PCOS and hypothyroidism (18). According to the same study, it was indicated that the TSH-lowering effect of metformin was more profound in individuals with more severe hypothyroidism. The lack of effect of metformin on TSH in our study might be explained by the fact that the majority of our participants was euthyroid.

A novel finding in the present study was the smaller decrease in fT4 during pregnancy by metformin compared to placebo. It has previously been suggested by Haddow et al. (2016) that low fT4 levels may serve as a surrogate marker for an increased peripheral deiodinase activity in conditions of obesity and insulin resistance (27). This will result in increased conversion of T4 to T3 and thus low levels of fT4 and higher T3/T4 ratio. Since increased peripheral deiodinase activity should also be present among PCOS women, we speculate that metformin could cause a suppression of this, resulting in higher levels of fT4 in the metformin group compared to placebo.

Regarding pregnancy outcomes, we did not find any association between the change in fT4 from inclusion to gw19 and the pregnancy complications preterm delivery, preeclampsia and GDM, independently of randomization group. However, the change in fT4 from the first to the second trimester tended to be associated with a lower risk of GDM. Several studies have reported inverse associations between fT4 and GDM. Most studies have found evidence for this association in the second and third trimester but not in the first (27–30). However, Yang et al. could demonstrate that even in early pregnancy low fT4 levels may predispose to GDM (31). In a cohort of 9351 euthyroid pregnant women, low fT4 was associated with GDM. The authors suggested that the causal factor contributing to GDM was the high levels of T3, caused by an increased deiodinase activity and indicated by low fT4 levels (27). This mechanism may also explain our results. Furthermore, the finding of the association between less weight gain during pregnancy and a smaller decrease in fT4 could indicate a link between insulin resistance and the lower fT4 levels. This is supported by the weak correlation between gestational weight gain and the change in fT4 during pregnancy in the whole cohort.

The strengths of our study include the randomized placebo-controlled, multi-center design, the longitudinal follow-up during pregnancy, the good adherence to study medication and the lack of inclusion limitations according to PCOS phenotypes or BMI. Nevertheless, there are certain limitations related to the present study. First, the estimated prevalence of hypothyroid disorders could not be related to a non-PCOS population since we lack a control group. However, this was beyond the aim of this RCT. Furthermore, T3 levels were not measured, and therefore we were not able to calculate the T3/T4 ratio, which is reported to be a measure of deiodinase activity. In addition, as we lack population- and trimester-specific reference ranges for TSH and fT4, we had to use the non-pregnant reference range for fT4.

In conclusion, we have demonstrated that fT4 decreased less and weight gain was lower in women with PCOS treated with metformin during pregnancy in comparison with placebo. The lower decrease in fT4 tended to be associated with a lower risk for GDM. The prevalence of mild hypothyroidism in the first trimester among pregnant women with PCOS was low in this study. Future studies are needed to confirm our results, as well as to increase our understanding of the underlying mechanisms for the possible effect of metformin on thyroid hormone status.

## Data Availability Statement

The original contributions presented in the study are included in the article/supplementary material. Further inquiries can be directed to the corresponding author.

## Ethics Statement

The studies involving human participants were reviewed and approved by Committee for Medical Research Ethics of Health Region IV (Midt-Norge), original studies 2001-02-01 and 2004-09-06, reference numbers 51-2000 and 145-04, current study 2015-02-19, reference number 2010/3115 145-04. The patients/participants provided their written informed consent to participate in this study.

## Author Contributions

EV conceived and designed the original protocol. On the initiative of AT and AH, the protocol of the present study was written together with EV. AT managed the database and ran preliminary analysis with input from AH and EV. AT, MA, AH, EV, and JC analyzed the data, and a statistician performed the statistical analyses. AT drafted the manuscript with input from AH, EV, MA, JC, and BÅ. All authors have full access to all data in the study and take responsibility for the integrity of the data and the accuracy of the data analysis. AT, AH, and EV are guarantors of the paper and affirm that the manuscript is an honest, accurate, and transparent account of the study being reported; that no important aspects of the study have been omitted; and that any discrepancies from the study as planned have been explained. All authors contributed to the article and approved the submitted version.

## Funding

This study was supported financially by the Swedish Research Council (AH 2017-02051), Karolinska Institutet, the regional agreement on medical training and clinical research between Stockholm County Council and Karolinska Institutet (AH) and the Centre for Clinical Research Sörmland, Uppsala University, Uppsala, Sweden (AT). The Liaison Committee between the Central Norway Regional Health Authority and the Norwegian University of Science and Technology was funding the PregMet study (EV). Weifa A/S, Oslo, Norway delivered metformin and placebo tablets free of charge.

## Conflict of Interest

The authors declare that the research was conducted in the absence of any commercial or financial relationships that could be construed as a potential conflict of interest.

## Publisher’s Note

All claims expressed in this article are solely those of the authors and do not necessarily represent those of their affiliated organizations, or those of the publisher, the editors and the reviewers. Any product that may be evaluated in this article, or claim that may be made by its manufacturer, is not guaranteed or endorsed by the publisher.

## References

[1] Bozdag G Mumusoglu S Zengin D Karabulut E Yildiz BO. The Prevalence and Phenotypic Features of Polycystic Ovary Syndrome: A Systematic Review and Meta-Analysis. Hum Reprod (Oxf Engl) (2016) 31(12):2841–55. doi: 10.1093/humrep/dew218 27664216

[2] Franks S. Polycystic Ovary Syndrome. N Engl J Med (1995) 333(13):853–61. doi: 10.1056/nejm199509283331307 7651477

[3] Novais Jde S Benetti-Pinto CL Garmes HM Jales RM Juliato CR. Polycystic Ovary Syndrome and Chronic Autoimmune Thyroiditis. Gynecol Endocrinol (2015) 31(1):48–51. doi: 10.3109/09513590.2014.958990 25211537

[4] Ding X Yang L Wang J Tang R Chen Q Pan J. Subclinical Hypothyroidism in Polycystic Ovary Syndrome: A Systematic Review and Meta-Analysis. Front Endocrinol (2018) 9:700. doi: 10.3389/fendo.2018.00700 PMC6277795 30542323

[5] Romitti M Fabris VC Ziegelmann PK Maia AL Spritzer PM. Association Between PCOS and Autoimmune Thyroid Disease: A Systematic Review and Meta-Analysis. Endocrine Connect (2018) 7(11):1158–67. doi: 10.1530/ec-18-0309 PMC6215798 30352422

[6] Qin JZ Pang LH Li MJ Fan XJ Huang RD Chen HY. Obstetric Complications in Women With Polycystic Ovary Syndrome: A Systematic Review and Meta-Analysis. Reprod Biol Endocrinol: RB&E (2013) 11:56. doi: 10.1186/1477-7827-11-56 23800002 PMC3737012

[7] Taylor PN Lazarus JH. Hypothyroidism in Pregnancy. Endocrinol Metab Clin North Am (2019) 48(3):547–56. doi: 10.1016/j.ecl.2019.05.010 31345522

[8] Abalovich M Gutierrez S Alcaraz G Maccallini G Garcia A Levalle O. Overt and Subclinical Hypothyroidism Complicating Pregnancy. Thyroid: Off J Am Thyroid Assoc (2002) 12(1):63–8. doi: 10.1089/105072502753451986 11838732

[9] Su PY Huang K Hao JH Xu YQ Yan SQ Li T. Maternal Thyroid Function in the First Twenty Weeks of Pregnancy and Subsequent Fetal and Infant Development: A Prospective Population-Based Cohort Study in China. J Clin Endocrinol Metab (2011) 96(10):3234–41. doi: 10.1210/jc.2011-0274 21832110

[10] Chen L Hu R. Thyroid Autoimmunity and Miscarriage: A Meta-Analysis. Clin Endocrinol (2011) 74(4):513–9. doi: 10.1111/j.1365-2265.2010.03974.x 21198746

[11] Negro R. Thyroid Autoimmunity and Pre-Term Delivery: Brief Review and Meta-Analysis. J Endocrinol Invest (2011) 34(2):155–8. doi: 10.1007/bf03347047 21422804

[12] Glintborg D Rubin KH Nybo M Abrahamsen B Andersen M. Increased Risk of Thyroid Disease in Danish Women With Polycystic Ovary Syndrome: A Cohort Study. Endocrine Connect (2019) 8(10):1405–15. doi: 10.1530/ec-19-0377 PMC6826171 31518989

[13] Jakubowicz DJ Iuorno MJ Jakubowicz S Roberts KA Nestler JE. Effects of Metformin on Early Pregnancy Loss in the Polycystic Ovary Syndrome. J Clin Endocrinol Metab (2002) 87(2):524–9. doi: 10.1210/jcem.87.2.8207 11836280

[14] Thatcher SS Jackson EM. Pregnancy Outcome in Infertile Patients With Polycystic Ovary Syndrome Who Were Treated With Metformin. Fertil Steril (2006) 85(4):1002–9. doi: 10.1016/j.fertnstert.2005.09.047 16580387

[15] Glueck CJ Wang P Goldenberg N Sieve-Smith L. Pregnancy Outcomes Among Women With Polycystic Ovary Syndrome Treated With Metformin. Hum Reprod (Oxf Engl) (2002) 17(11):2858–64. doi: 10.1093/humrep/17.11.2858 12407039

[16] Lovvik TS Carlsen SM Salvesen O Steffensen B Bixo M Gomez-Real F. Use of Metformin to Treat Pregnant Women With Polycystic Ovary Syndrome (PregMet2): A Randomised, Double-Blind, Placebo-Controlled Trial. Lancet Diabetes Endocrinol (2019) 7(4):256–66. doi: 10.1016/s2213-8587(19)30002-6 30792154

[17] Morteza Taghavi S Rokni H Fatemi S. Metformin Decreases Thyrotropin in Overweight Women With Polycystic Ovarian Syndrome and Hypothyroidism. Diabetes Vasc Dis Res (2011) 8(1):47–8. doi: 10.1177/1479164110391917 21262871

[18] Rotondi M Cappelli C Magri F Botta R Dionisio R Iacobello C. Thyroidal Effect of Metformin Treatment in Patients With Polycystic Ovary Syndrome. Clin Endocrinol (2011) 75(3):378–81. doi: 10.1111/j.1365-2265.2011.04042.x 21521311

[19] Vanky E Salvesen KA Heimstad R Fougner KJ Romundstad P Carlsen SM. Metformin Reduces Pregnancy Complications Without Affecting Androgen Levels in Pregnant Polycystic Ovary Syndrome Women: Results of a Randomized Study. Hum Reprod (Oxf Engl) (2004) 19(8):1734–40. doi: 10.1093/humrep/deh347 15178665

[20] Vanky E Stridsklev S Heimstad R Romundstad P Skogoy K Kleggetveit O. Metformin Versus Placebo From First Trimester to Delivery in Polycystic Ovary Syndrome: A Randomized, Controlled Multicenter Study. J Clin Endocrinol Metab (2010) 95(12):E448–55. doi: 10.1210/jc.2010-0853 20926533

[21] Rotterdam ESHRE/ASRM-Sponsored PCOS Consensus Workshop Group. Revised 2003 Consensus on Diagnostic Criteria and Long-Term Health Risks Related to Polycystic Ovary Syndrome (PCOS). Rotterdam ESHRE/ASRM-Sponsored PCOS Consensus Workshop Group. Hum Reprod (Oxf Engl) (2004) 19(1):41–7. doi: 10.1093/humrep/deh098 14688154

[22] Alexander EK Pearce EN Brent GA Brown RS Chen H Dosiou C. Guidelines of the American Thyroid Association for the Diagnosis and Management of Thyroid Disease During Pregnancy and the Postpartum. Thyroid: Off J Am Thyroid Assoc (2017) 27(3):315–89. doi: 10.1089/thy.2016.0457 28056690

[23] Altman DG. Practical Statistics for Medical Research. London: Chapman & Hall (1999).

[24] Vanky E DEZ F Diaz M Ibanez L Carlsen SM. On the Potential of Metformin to Prevent Preterm Delivery in Women With Polycystic Ovary Syndrome - an Epi-Analysis. Acta Obstet Gynecol Scand (2012) 91(12):1460–4. doi: 10.1111/aogs.12015 23006146

[25] Karcaaltincaba D Ozek MA Ocal N Calis P Inan MA Bayram M. Prevalences of Subclinical and Overt Hypothyroidism With Universal Screening in Early Pregnancy. Arch Gynecol Obstet (2020) 301(3):681–6. doi: 10.1007/s00404-020-05462-0 32107608

[26] Lupoli R Di Minno A Tortora A Ambrosino P Lupoli GA Di Minno MN. Effects of Treatment With Metformin on TSH Levels: A Meta-Analysis of Literature Studies. J Clin Endocrinol Metab (2014) 99(1):E143–8. doi: 10.1210/jc.2013-2965 24203069

[27] Haddow JE Craig WY Neveux LM Palomaki GE Lambert-Messerlian G Malone FD. Free Thyroxine During Early Pregnancy and Risk for Gestational Diabetes. PloS One (2016) 11(2):e0149065. doi: 10.1371/journal.pone.0149065 26910563 PMC4766100

[28] Cleary-Goldman J Malone FD Lambert-Messerlian G Sullivan L Canick J Porter TF. Maternal Thyroid Hypofunction and Pregnancy Outcome. Obstet Gynecol (2008) 112(1):85–92. doi: 10.1097/AOG.0b013e3181788dd7 18591312 PMC4949950

[29] Oguz A Tuzun D Sahin M Usluogullari AC Usluogullari B Celik A. Frequency of Isolated Maternal Hypothyroxinemia in Women With Gestational Diabetes Mellitus in a Moderately Iodine-Deficient Area. Gynecol Endocrinol (2015) 31(10):792–5. doi: 10.3109/09513590.2015.1054801 26190538

[30] Olivieri A Valensise H Magnani F Medda E De Angelis S D’Archivio M. High Frequency of Antithyroid Autoantibodies in Pregnant Women at Increased Risk of Gestational Diabetes Mellitus. Eur J Endocrinol/Eur Fed Endocrine Soc (2000) 143(6):741–7. doi: 10.1530/eje.0.1430741 11124856

[31] Yang S Shi FT Leung PC Huang HF Fan J. Low Thyroid Hormone in Early Pregnancy Is Associated With an Increased Risk of Gestational Diabetes Mellitus. J Clin Endocrinol Metab (2016) 101(11):4237–43. doi: 10.1210/jc.2016-1506 27583471

